# Time-Course Metabolomic Analysis: Production of Betaine Structural Analogs by Fungal Fermentation of Seaweed

**DOI:** 10.3390/metabo14040201

**Published:** 2024-04-03

**Authors:** Nao Inoue, Keisuke Tsuge, Teruyoshi Yanagita, Akira Oikawa, Koji Nagao

**Affiliations:** 1 Department of Biological Resource Science, Saga University, 1 Honjo-machi, Saga 840-8502, Japan; d5589@cc.saga-u.ac.jp (N.I.); yanagitt@cc.saga-u.ac.jp (T.Y.); 2 The United Graduate School of Agricultural Sciences, Kagoshima University, Kagoshima 890-0065, Japan; 3 Saga Regional Industry Support Center, Saga 849-0932, Japan; tsuge@saga-itc.jp; 4 Graduate School of Agriculture, Kyoto University, Kyoto 606-8502, Japan; oikawa.akira.7j@kyoto-u.ac.jp

**Keywords:** stachydrine, betaine, carnitine, *Aspergillus oryzae*, *Neopyropia yezoensis*, fungal fermentation, seaweed

## Abstract

Betaine structural analogs are compounds characterized by the presence of positive and negative charges in a single molecule and have been reported to have physiological properties, such as anti-inflammatory activities. In this study, we performed a metabolomic analysis of metabolite composition changes during the fermentation of *Neopyropia yezoensis*, an edible red alga, with *Aspergillus oryzae* for 72 h. The results indicated that three specific betaine structural analogs (betaine, stachydrine, and carnitine) exhibited significant changes in production by the end of the 72 h fermentation period. Time-course analysis suggested that betaine was generated from the precursor choline at 12–24 h during the late stage of fungal growth, while stachydrine was generated from the precursor-related compound glutamic acid at 48–72 h during the sporulation stage. However, the contribution of the precursor lysine to the increased production of carnitine during the 12–72 h period was unclear. This study provides useful information on the efficient production of betaine structural analogs by the fungal fermentation of seaweed as well as various other food materials.

## 1. Introduction

Betaine is a generic term for a neutral compound with a positively charged cationic functional group without hydrogen atoms and a negatively charged functional group that may not be adjacent to a cationic moiety. Stachydrine, a type of alkaloid classified as a pyrrolidine derivative and also referred to as proline betaine, is one of the major components of the medicinal plant *Leonurus japonicus Houtt* (also known as Motherwort or Yi Mu Cao). This plant has been used as a traditional medicine in Asia to treat menoxenia, dysmenorrhea, amenorrhea, lochia, body edema, oliguresis, sores, ulcerations, and other disorders in women [1,2]. Recent pharmacological studies have also shown that this medicinal herb may act on the cardiovascular system, brain tissue, and central nervous system [1,2,3,4]. Similarly, stachydrine has been reported to have diverse pharmacological actions, including cardioprotective, antioxidant, anti-inflammatory, neuroprotective, and anticancer effects, and is assumed to be an efficient therapeutic agent against cardiovascular diseases and cancer [1,2,3,4]. Betaine (also known as glycine betaine), an intramolecular salt with quaternary ammonium as the cation and carboxylic acid as the anion, was named after its discovery in sugar beets (*Beta vulgaris* subsp. *vulgaris*). Betaine, a type of alkaloid, is widely distributed not only in plants but also in animals and microorganisms and plays important roles in various physiological activities as an osmoprotectant and methyl group donor in the human body [5,6]. The preventive and therapeutic effects of betaine administration on liver diseases have been extensively studied [7,8]. Furthermore, betaine is expected to have potentially beneficial effects on various human diseases through its anti-inflammatory, antioxidant, anti-endoplasmic reticulum stress, and antitumor effects [5,6,7,8]. Carnitine, also known as lysine betaine, is an intramolecular salt with quaternary ammonium as a cation and carboxylic acid as an anion and is a vitamin-like compound that regulates lipid metabolism and energy production [9,10,11,12]. Carnitine homeostasis is primarily regulated by dietary intake and biosynthesis in the liver and other organs, although secondary deficiency is a concern in patients with organ disorders such as cirrhosis and Alzheimer’s disease [9,10,11,12]. In addition, L-carnitine supplementation has potential therapeutic effects on liver diseases, such as non-alcoholic fatty liver disease, non-alcoholic steatohepatitis, cirrhosis, and hepatic cancer [9,10,11,12].

Fermentation is a metabolic process in which enzymes cause chemical changes in organic compounds. It involves microorganisms breaking down organic macromolecules, such as carbohydrates and proteins, to obtain energy and simultaneously produce various byproducts that are beneficial to humans. That is, the fermentation of foods can impart tastes, umami, and aromas that are not found in the original ingredients and may also add health benefits [13]. *Aspergillus oryzae*, also known as yellow *koji* mold, is a filamentous fungus used in Japan to saccharify rice, sweet potatoes, and barley, to produce alcoholic beverages, such as sake and shochu, and to ferment soybeans to produce soy sauce and miso [14,15,16]. Although rare, seaweeds have been used in lactic acid fermentation and fungal fermentation [17,18,19]. Previously, we conducted fungal fermentation of “Nori”, a sheet of edible red algae *Neopyropia yezoensis* used in Japanese cuisine to prepare sushi and rice balls, to release the contained eicosapentaenoic acid (EPA) by breaking down the cell walls [20]. In our previous study, we demonstrated, for the first time, that three kinds of betaine structural analogs were produced by fungal fermentation of seaweed and that obese mice that ingested the fermented Nori powder exhibited dramatically improved fatty liver. The anti-inflammatory properties of EPA and betaine structural analogs contained in fermented Nori powder were assumed to have suppressed chronic inflammation in obese *db*/*db* mice, thereby demonstrating their functionality [20].

It is currently unclear how betaine structural analogs are produced during the fungal fermentation of seaweed. To investigate the timing of the production of these useful metabolites and their relationship with other nutrients involved in their production, metabolomic analysis was performed to evaluate the changes in the metabolite composition during the fermentation of *Neopyropia yezoensis*, an edible seaweed, with *Aspergillus oryzae* for 72 h.

## 2. Materials and Methods

### 2.1. Sample Preparation

Nori sheets, a hot-air-dried form of the seaweed *Neopyropia yezoensis*, were supplied by JA Saga (Saga, Japan) and shredded prior to fungal fermentation with *Aspergillus oryzae*. Fungal fermentation of shredded Nori was performed according to the method described by Uchida et al. [19], with slight modifications. Three commercial products, namely Three Dia, Takara-kin, and Diamond C, containing *Aspergillus oryzae* strains, were purchased from Higuchi Moyashi Co. (Osaka, Japan) and mixed at a 2:2:1 ratio to yield a seed culture mixture. The seed culture mixture (0.5 g) and distilled water (50 mL) were added to the shredded Nori (50 g), mixed well, spread on a tray, wrapped with filter cloth, and conditioned at 30.5 °C and >90% humidity for 3 d using a fermenter (PF110D, Japan Kneader Co., Ltd., Kanagawa, Japan). Samples were photographed using a smartphone and collected before and after fermentation at 6, 12, 24, 48, and 72 h. Collected samples were lyophilized, powdered using a milling machine (TML162, Tescom Denki Co., Ltd., Tokyo, Japan), and stored at −80 °C until further analysis.

### 2.2. Capillary Electrophoresis Mass Spectrometry (CE MS)

Sample powders (10 mg, *n* = 3 at each time point) were suspended in 500 µL methanol containing 8 µM internal standards (methionine sulfone for cationic and camphor 10-sulfonic acid for anionic analyses), 500 µL chloroform, and 200 µL Milli-Q water, and subsequently centrifuged (20,400× *g*, 3 min, 4 °C). The upper layer of each solution was transferred to a 1.5 mL test tube, evaporated for 30 min using a centrifugal concentrator, and then separated into two layers. The upper layer was centrifugally filtered through a PALL Nanosep 3 kD cutoff filter at 9100× *g* at 4 °C. Thereafter, the filtrate was dried with the centrifugal concentrator. The residue was dissolved in 20 µL Milli-Q water containing 200 μM internal standards (3-aminopyrrolidine for cationic and trimesic acid for anionic analyses). The CE MS system and conditions were set as described by Oikawa et al. [21]. All CE-time-of-flight (TOF) MS experiments were performed using an Agilent G7100A CE Instrument (Agilent Technologies, Santa Clara, CA, USA), Agilent G6224A TOF LC/MS system, Agilent 1200 Infinity series G1311C Quad Pump VL, and the G1603A Agilent CE MS adapter and G1607A Agilent CE-electrospray ionization (ESI) MS sprayer kit. The G1601BA 3D-CE ChemStation Ver. B.04.03 software for CE and G3335-64002 MH Workstation were used. Separations were performed using a fused silica capillary (50 μm i.d. × 100 cm total length) filled with the following electrolytes: 1 M formic acid for cationic analyses or with 20 mM ammonium formate (pH 10.0) for anionic analyses as the electrolyte. Sample solutions were injected at 50 mbar for 15 s (15 nL). Prior to each run, the capillary was flushed with electrolyte for 5 min. The applied voltage was set to 30 kV. The capillary temperature was maintained at 20 °C, and the sample tray was cooled to below 4 °C. Approximately 50% (*v*/*v*) methanol/water containing 0.5 μM reserpine was delivered as the sheath liquid at a rate of 10 μL/min. ESI-TOF MS was conducted in the positive ion mode for cationic analyses or in the negative ion mode for anionic analyses, and the capillary voltage was set at 4 kV. The flow rate of the heated dry nitrogen gas (heater temperature, 300 °C) was maintained at 10 psig. In the TOF MS, the fragmentor, skimmer, and Oct RFV voltages were set at 110, 50, and 160 V for cationic analyses and at 120, 60, and 220 V for anionic analyses, respectively. Each acquired spectrum was automatically recalibrated using the reference masses of the reference standards. The methanol dimer ion ([2M + H]^+^, *m*/*z* = 65.0597) and reserpine ([M + H]^+^, *m*/*z* = 609.2806) for cationic analyses or the formic acid dimer ion ([2M − H]^−^, *m*/*z* = 91.0037) and reserpine ([M − H]^−^, *m*/*z* = 607.2661) for anionic analyses provided the lock mass for the accurate mass measurements. The accurate mass data were acquired at a rate of 1.5 cycles/s in the range of 50–1000 *m*/*z*. Metabolites were identified and quantified as described by Oikawa et al. [21].

### 2.3. Statistical Analysis

The quantified values were converted to z-scores and subjected to principal component analysis (PCA) and hierarchical cluster analysis (HCA) using MetaboAnalyst 5.0 “https://www.metaboanalyst.ca/ (accessed on 6 March 2024)”. Data values were also expressed as the mean ± standard error. To assess the differences among the six groups, data were analyzed by one-way analysis of variance (ANOVA), and all differences were analyzed by Tukey’s honest significant difference (HSD) post-hoc test using the StatPlus:mac Pro Version 8.0.1 (AnalystSoft Inc., Walnut, CA, USA) software. Differences were considered statistically significant at *p* < 0.05.

## 3. Results

### 3.1. Metabolomic Changes in Nori Powder during Fungal Fermentation

**Figure 1:**
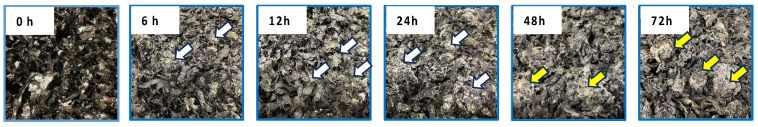
Appearance of seaweed before (0 h) and during fermentation by *Aspergillus oryzae* at 6, 12, 24, 48, and 72 h. White arrows indicate representative mycelial reproductive areas, and yellow arrows indicate representative spore-forming areas.

As shown in Figure 1, during the fermentation process, the Nori surface was initially covered with mycelia and turned white (6–24 h), followed by the formation of yellowish-brown colored spores (48–72 h).

**Figure 2:**
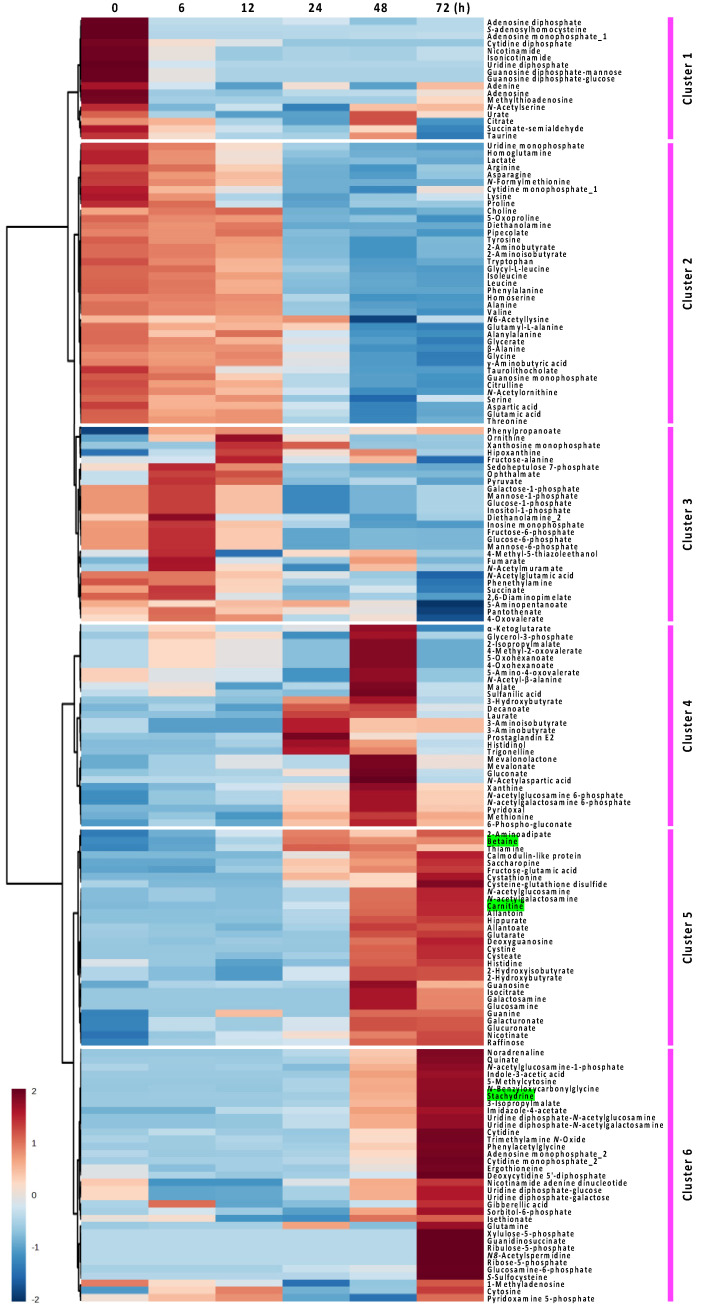
Hierarchical cluster analysis heatmaps of the 176 metabolites modified by fungal fermentation of Nori powder for 72 h. The red and blue colors correspond to high and low relative metabolite levels, respectively. Depending on the variation patterns of metabolite amounts, detected metabolites were classified into six clusters. Betaine structural analogs (betaine, carnitine, and stachydrine) are highlighted in green.

Further, we performed time-course metabolomic analyses to examine the differences in metabolites during the fungal fermentation of Nori powder. One hundred and seventy-six metabolites were identified and quantified using CE-TOF MS, and changes in the metabolome of fermented Nori powder were classified using HCA (Figure 2). The HCA heatmap revealed that the compounds in clusters 1–3 decreased during the 0–24 h fungal growth phase, suggesting that amino acids, nucleic acids, and nucleotides were consumed during primary metabolism. Metabolites (such as intermediates of the glycolytic pathway and citric acid cycle) in cluster 3 temporarily increased at 6–12 h and were subsequently consumed. These variations appeared to be related to the status of fungal growth during the fermentation process. In contrast, metabolites in clusters 4–6 (such as amines and gluconeogenesis precursors) were suggested to be produced as secondary metabolites during the late growth and sporulation phases (24–72 h). It is also interesting to note the behavior of the metabolites in cluster 4 (such as isoprenoid precursors and medium-chain fatty acids), which are produced during the early stages of sporulation at 24–48 h and then start to decline.

The fold-changes in metabolite levels relative to the 0 h time point (or appearance time point) were calculated, and the changes after 6, 12, 24, and 72 h are shown in Table 1. Among the 176 metabolites detected, 31 were newly produced during fungal fermentation, 49 metabolites were more than doubled at 72 h compared to that at the 0 h time point (or appeared time point), 53 metabolites were reduced to less than half at 72 h compared to that at the 0 h time point (or appeared time point), and 12 metabolites were lost during fungal fermentation. 

**Figure 3:**
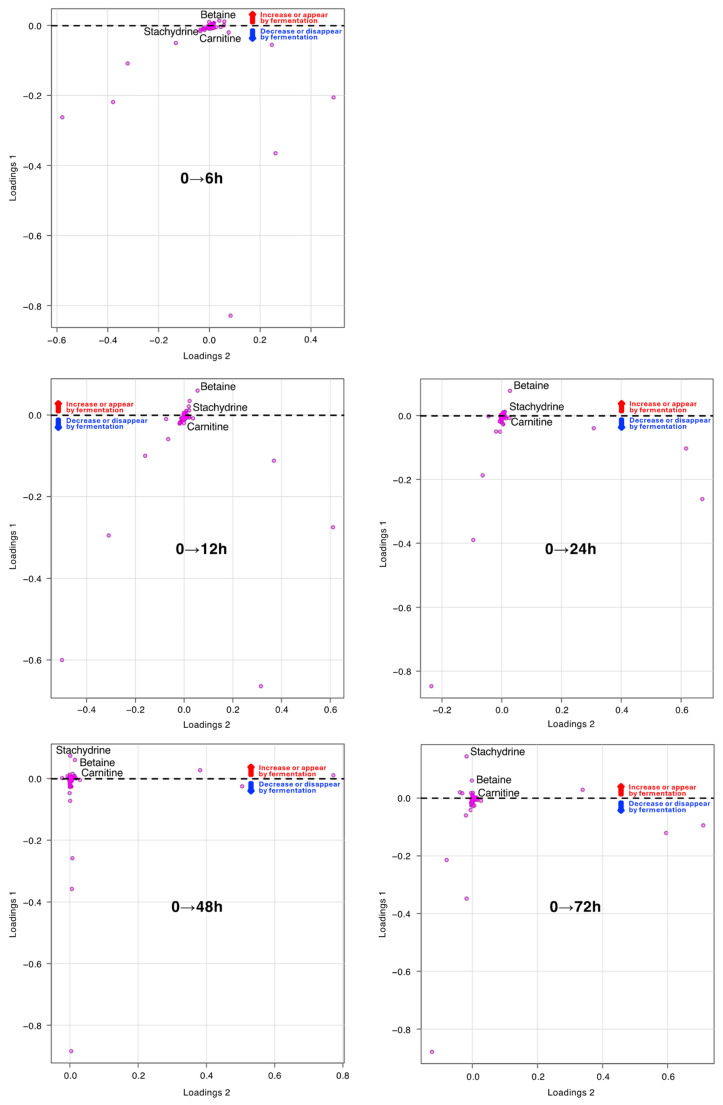
Principal components analysis loading plot of 176 metabolites from Nori powders before and after fungal fermentation for 6, 12, 24, 48, and 72 h. Metabolites with large quantitative variations are plotted above and below the dotted reference line.

As shown in Table 1, among the 176 metabolites, the top three metabolites in terms of fold-change in production at the end of 72 h of fermentation were betaine, stachydrine, and carnitine. Furthermore, loading plots of the PCA revealed that betaine production was most prominent at 12–24 h, and stachydrine production was most prominent at 48–72 h, in terms of the product volume increase among the 176 metabolites during the fungal fermentation process (Figure 3).

### 3.2. Changes in Stachydrine Production and Related Metabolite Levels

**Figure 4:**
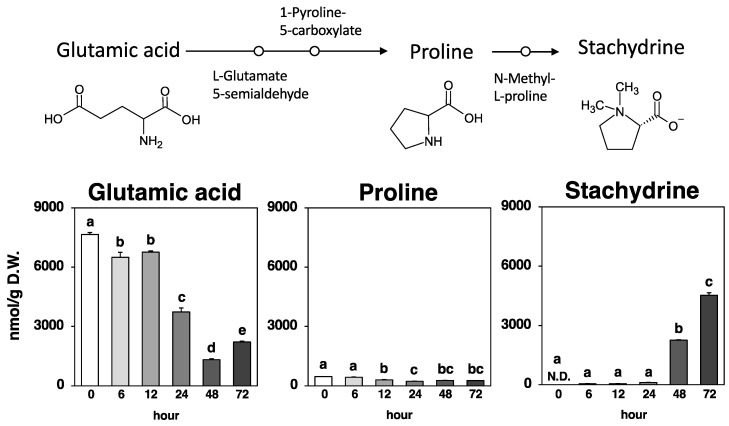
Stachydrine biosynthetic pathway and amounts of glutamic acid, proline, and stachydrine at each time point. Values are expressed as the mean ± standard error (*n* = 3). N.D.: not detected. Different letters on the bars denote significant differences (*p* < 0.05) among the six time points.

As shown in Figure 4, stachydrine, also known as (2S)-1,1-dimethylpyrrolidinium-2-carboxylate, which is derived by methylating the amino acid proline, was not detected in the Nori powder prior to fungal fermentation. Stachydrine production due to fungal fermentation was detected from 6 h, and after 72 h, it was the highest produced metabolite (from 0.0529 µmol/g at 6 h to 4.52 µmol/g at 72 h).

Proline, the precursor amino acid of stachydrine, exhibited only negligible quantitative variation (0.231–0.470 µmol/g) compared to stachydrine production (0–4.52 µmol/g). Meanwhile, glutamic acid, the precursor amino acid of proline, exhibited significant variation (1.32–7.66 µmol/g) comparable to the production of stachydrine. That is, a dramatic decrease (3.02 µmol/g) occurred during the 12–24 h period, prior to the 48 h time point when significant stachydrine production began, and an even more pronounced decrease (2.42 µmol/g) during the 24–48 h period (Figure 4).

### 3.3. Changes in Betaine Production and Related Metabolite Levels

**Figure 5:**
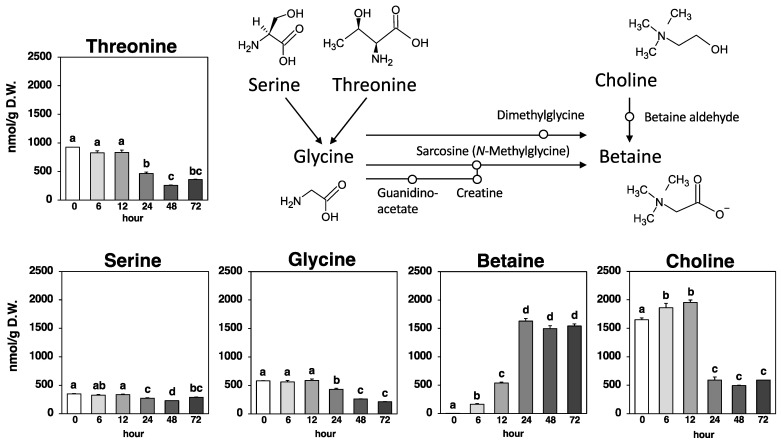
Betaine biosynthetic pathway and amounts of threonine, serine, glycine, betaine, and choline at each time point. Values are expressed as the mean ± standard error (*n* = 3). Different letters on the bars denote significant differences (*p* < 0.05) among the six time points.

As shown in Figure 5, betaine, known as trimethylammonioacetate, which is derived by methylating the amino acid glycine, was slightly detected (0.00245 µmol/g) in Nori powder prior to fungal fermentation. The amount of betaine in the fungus-fermented Nori increased exponentially up to 24 h (to 1.63 µmol/g) and then remained steady until 72 h (1.50–1.63 µmol/g). 

Glycine, the putative precursor amino acid of betaine, and serine and threonine, the precursor amino acids of glycine, exhibited variable amounts (0.432–0.588 µmol/g, 0.271–0.348 µmol/g, and 0.466–0.924 µmol/g, respectively) that may have partially contributed to betaine production during the 0–24 h period. On the other hand, another pathway was present that produced betaine through a two-step oxidation reaction, utilizing choline as a precursor, and the marked increase (1.09 µmol/g) in betaine during the 12–24 h period was consistent with the significant decrease (1.36 µmol/g) in choline during the same time period (Figure 5).

### 3.4. Changes in Carnitine Production and Related Metabolite Levels

**Figure 6:**
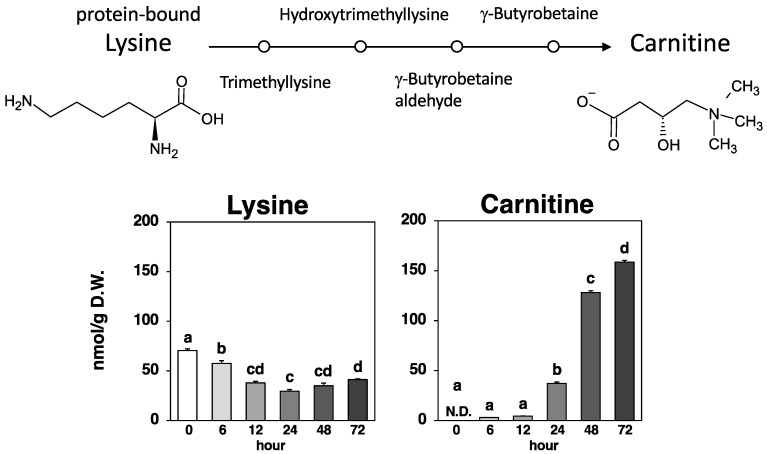
Carnitine biosynthetic pathway and amounts of lysine and carnitine at each time point. Values are expressed as the mean ± standard error (*n* = 3). N.D.: not detected. Different letters on bars denote significant differences (*p* < 0.05) among the six time points.

As shown in Figure 6, carnitine, also known as γ-trimethyl-β-oxybutyrobetain, which is derived by methylating the amino acid lysine, was not detected in Nori powder prior to fungal fermentation. Carnitine production due to fungal fermentation was detected after 6 h, and after 72 h, it was one of the significantly produced metabolites by the fungal fermentation of Nori (from 0.00297 µmol/g at 6 h to 0.159 µmol/g at 72 h). 

Lysine, which provides the carbon backbone to carnitine, exhibited a variable amount (0.0295–0.0705 µmol/g) that may have partially contributed to the marked increase in carnitine production (0.156 µmol/g) during the 6–72 h period; however, the direct relationship is unclear.

## 4. Discussion

Secondary metabolism occurs in many organisms, including plants, bacteria, and fungi, and facilitates the production of compounds that are not essential for organism growth, though they may have various biological activities that allow organisms to grow advantageously. Fungi are known to biosynthesize various secondary metabolites, some of which are harmful to humans, such as mycotoxins, while some are beneficial to humans, such as antibiotics [22]. We previously observed that fungus-fermented Nori powder contains betaine structural analogs, such as stachydrine, betaine, and carnitine, which exhibit hepatoprotective effects in obese *db*/*db* mice [20]. In this study, we attempted a dynamic approach using time-course metabolomic analysis to provide an overview of the production of betaine structural analogs by fungal fermentation of seaweed. The production of secondary metabolites is known to begin in the late stages of microbial growth and is associated with sporulation [22]; sporulation was observed during the first 24–48 h of fungal fermentation of seaweed in the present experiment (Figure 1). Based on the results of the time-course analysis using CE-TOF MS, we found that stachydrine was mainly produced at 48–72 h during the sporulation stage (Figure 4), betaine was mainly produced at 12–24 h during the late stage of fungal growth (Figure 5), and carnitine was mainly produced at 12–72 h during the stages of late growth to sporulation (Figure 6). The final production volume of the three betaine structural analogs after the 72 h fermentation of Nori using *Aspergillus oryzae* was in the following order: stachydrine (4.52 µmol/g) > betaine (1.63 µmol/g) > carnitine (0.159 µmol/g). In our previous study, fermented Nori powder provided by Maruhide Shoyu Co. contained 9.80, 7.75, and 1.14 µmol/g of stachydrine, betaine, and carnitine, respectively [20]. This suggests that the production of betaine structural analogs by fungal fermentation can be improved if fermentation temperature and humidity are strictly regulated using more appropriate equipment [19].

Stachydrine (proline betaine) is found in many plants, such as Motherwort, citrus herbs and fruits, chestnuts, and alfalfa [1,2,3,4,23]. The injection of proline into hollow stems has been reported to increase stachydrine content in plants [24]. Our results revealed that variations in proline content seemed to have less effect on stachydrine production during fungal fermentation (Figure 4). Two pathways have been postulated for the formation of proline: one starting with ornithine and the other starting with glutamic acid, although the former is not important in animals [24]. Our results also indicated that ornithine exhibited negligible quantitative variation (0.009–0.021 µmol/g), and did not seem to contribute to stachydrine production (0–4.52 µmol/g, Figure 4). Thus, we assumed that stachydrine was produced from glutamic acid via pyrrolidonecarboxylic acid, proline, and methylproline through fungal fermentation (Figure 4). Stachydrine production by fermentation was described in a previous paper that examined the effect of the production process (traditional or modern methods) of “*Doenjang*”, a traditional Korean fermented soybean-based food, on metabolite formation [25]. The results suggested that stachydrine is abundant when traditional production methods are used and that the level of metabolites varies significantly depending on the microbial community, including *Aspergillus oryzae* [25]. Another paper on fermentation and stachydrine concerns “*Makgeolli*”, a traditional Korean alcoholic beverage brewed from rice [26]. One of the 58 types of *Makgeolli* brewed with various *Nurks* (fermentation starters) has an anti-obesity effect, and stachydrine is responsible for that effect [26]. Although this study did not analyze the microflora that contributed to the fermentation of stachydrine-containing *Makgeolli* was not conducted [26], a report suggested that *Aspergillus oryzae* can be found in several *Nurks* used in Korea [27]. Given that stachydrine is produced during the fermentation of seaweed (Nori), soybeans (*Doenjang*), and rice (*Makgeolli*), the involvement of *Aspergillus oryzae* in fermentation, rather than food materials, may be most important for the efficient production of stachydrine. 

Betaine, a glycine derivative with three methyl groups, is produced endogenously through choline metabolism or exogenously ingested via the human diet [5]. The results of this experiment suggested that *Aspergillus oryzae* also uses choline in seaweed as a precursor for significant betaine production (Figure 5). Cereals, vegetables, and seafood are considered the major sources of dietary betaine, which is recognized as a GRAS (Generally Recognized as Safe) ingredient. Since various functionalities such as antioxidant, hepatoprotective, neuroprotective, myocardial protective, and sports ability-enhancing effects of betaine supplementation have been reported [5,6,7,8], betaine production using fungal fermentation may be a promising approach to add health functions to foods.

Carnitine (lysine betaine, L-carnitine) is an essential water-soluble molecule with multiple functions in the human body, and its homeostasis is primarily regulated by dietary intake and biosynthesis in the liver and other organs [9,10,11,12]. The major sources of L-carnitine are red meat, which is consumed by adults, and milk, which is consumed by infants and children [11,12]. Since plants contain only trace amounts, strict vegetarians obtain more than 90% of their requirements through endogenous synthesis [11,12]. The synthesis of L-carnitine starts with trimethyllysine, which is released through the degradation of lysosomal proteins and ends with the hydroxylation of γ-butyrobetaine (BB) by γ-butyrobetaine dioxygenase (BBD) to produce endogenous carnitine. As shown in Figure 6, the results of this experiment did not reveal a clear relationship between the variable amounts of lysine (starting material) and carnitine production (final material). A more definite relationship might be observed by examining the amount of BB, which is a direct precursor of carnitine. The final enzyme (BBD) in the synthetic pathway is present in the liver, kidneys, testes, and brain; therefore, L-carnitine is produced only in these organs. The yeast *Saccharomyces cerevisiae* is known to be unable to synthesize carnitine endogenously [28], whereas the fungus *Aspergillus oryzae* has been reported to exhibit high L-carnitine production capacity in immersion culture [29]. Carnitine production through fungal fermentation of plant materials may have potential as a countermeasure against secondary deficiencies due to organ damage [9,10] and could be introduced into vegetarian diets [11,12].

In this study, marked decreases in compounds required for fungal growth were observed during 0–24 h (clusters 1–3, Figure 2). Whereas the characteristic production of several metabolites other than betaine structural analogs was also observed in secondary metabolism during 24–72 h (clusters 4–6, Figure 2). Allantoin, a derivative of urea and also one of the essential compounds in many herbs, has been reported to have wound-healing and anti-inflammatory properties [30]. Allantoin was detected (0.0126 µmol/g) in Nori powder prior to fungal fermentation, and its production was most significant at 24–72 h (from 0.070 µmol/g at 24 h to 0.476 µmol/g at 72 h) during the stages of late growth to sporulation (Figure 2 and Table 1). Ergothioneine is a thiol/thione molecule synthesized only by some fungi and bacteria and has been reported to have antioxidant, anti-inflammatory, and anti-neurodegenerative properties [31]. Ergothioneine was detected (1.00 µmol/g) in Nori powder prior to fungal fermentation, and after 72 h, it was one of the most abundant metabolites (1.51 µmol/g) in the fungus-fermented Nori powder (Figure 2 and Table 1). Trigonelline, the main alkaloid isolated from the medicinal plant fenugreek, has been shown to have therapeutic effects in diabetes and neurological diseases [32]. Trigonelline was not detected in Nori powder prior to fungal fermentation; however, it was detected after 24 h (0.004 µmol/g) and then subsequently decreased until 72 h (0.004–0.001 µmol/g) (Figure 2 and Table 1).

## 5. Conclusions

Time-course analysis suggested that betaine was generated from the precursor choline at 12–24 h during the late stage of fungal growth, while stachydrine was generated from the precursor-related compound glutamic acid at 48–72 h during the sporulation stage. Although the contribution of the precursor lysine was unclear, carnitine was generated at 12–72 h during the late growth and sporulation stages. 

In this study, *Aspergillus oryzae* (yellow *koji* mold) was used for seaweed fermentation to produce betaine structural analogs. In Japan, various fungi belonging to the genus *Aspergillus* have been used for traditional food fermentation, including *Aspergillus luchuensis* (black *koji* mold used for Awamori Shochu production) [33] and *Aspergillus luchuensis* mut. *Kawachii* (white *koji* mold for Honkaku Shochu production) [33] and *Aspergillus sojae* (soy sauce *koji* mold for Shoyu production) [15]. It would be interesting to examine whether different combinations of various *koji* molds *(Aspergillus* spp.) and foodstuffs (seaweed, rice, wheat, soybeans, etc.) will affect the production of betaine structural analogs in future studies.

This study provides useful information on the efficient production of betaine structural analogs using the fungal fermentation of seaweed as well as various other food materials.

## Figures and Tables

**Table 1:** The fold-changes in metabolite levels relative to the 0 h time point (or appearance time point) in Nori powder during fungal fermentation for 72 h. Metabolites that have a fold change > 2.0 (red) and <0.5 (blue) are shown. Metabolites are listed from the highest to lowest fold changes at 72 h of fermentation (the top three metabolites at 72 h are highlighted in yellow).

Metabolite	6 h	12 h	24 h	48 h	72 h	Metabolite	6 h	12 h	24 h	48 h	72 h
Betaine	65.80	219.75	665.76	611.34	630.32	4-Methyl-5-thiazole-ethanol	1.26	0.78	1.06	1.11	0.93
Stachydrine	Appear	1.02	2.12	42.58	85.38	*N*-Acetylserine	0.82	0.86	0.78	0.92	0.92
Carnitine	Appear	1.50	12.50	43.10	53.36	Urate	0.86	0.82	0.82	1.01	0.92
Quinate	1.20	1.01	2.22	18.19	41.84	*N*-Acetyl-β-alanine	0.95	0.93	0.81	1.20	0.87
Allantoin	1.05	1.11	5.52	30.87	37.76	5-Amino-4-oxovalerate	0.95	0.93	0.81	1.20	0.87
Uridine diphosphate-*N*-acetylgalactosamine	1.07	1.47	5.67	18.04	33.04	Serine	0.93	0.97	0.78	0.66	0.83
Uridine diphosphate-*N*-acetylglucosamine	1.07	1.47	5.67	18.04	33.04	Fructose-alanine	0.99	1.22	1.00	1.09	0.81
Nicotinate	7.07	9.85	12.78	18.60	21.18	2-Isopropylmalate	1.26	1.15	0.87	1.91	0.80
Cytidine monophosphate_2	n.d.	n.d.	Appear	4.91	18.18	4-Methyl-2-oxovalerate	1.26	1.15	0.87	1.91	0.80
3-Isopropylmalate	n.d.	n.d.	Appear	8.48	17.75	4-Oxohexanoate	1.26	1.15	0.87	1.91	0.80
5-Methylcytosine	n.d.	n.d.	Appear	7.39	13.73	5-Oxohexanoate	1.26	1.15	0.87	1.91	0.80
*S*-Sulfocysteine	n.d.	n.d.	n.d.	Appear	12.54	Adenine	0.61	0.44	0.67	0.44	0.75
Thiamine	2.62	5.89	13.97	13.23	10.18	Diethanolamine_2	1.40	0.84	0.81	0.61	0.75
Noradrenaline	n.d.	n.d.	Appear	4.06	9.72	Taurine	0.87	0.81	0.81	0.94	0.72
Xanthine	2.72	6.15	8.00	19.53	9.43	*N6*-Acetyllysine	0.92	1.03	1.09	0.19	0.70
Glucosamine-6-phosphate	n.d.	n.d.	Appear	1.54	9.21	Isocitrate	n.d.	n.d.	n.d.	Appear	0.70
Adenosine monophosphate_2	0.39	0.45	0.79	3.28	9.06	Glucosamine	n.d.	n.d.	n.d.	Appear	0.70
*N*-Benzyloxycarbonylglycine	1.14	0.97	1.54	5.01	8.59	Galactosamine	n.d.	n.d.	n.d.	Appear	0.70
Glutamine	1.27	1.28	5.23	0.62	8.59	Lysine	0.81	0.54	0.42	0.50	0.58
Guanosine	3.21	3.47	2.86	13.98	8.14	Proline	0.91	0.64	0.49	0.57	0.57
Saccharopine	n.d.	Appear	4.73	5.82	8.08	Cytidine monophosphate_1	0.69	0.53	0.28	0.17	0.55
Cysteine-glutathione disulfide	Disappear	n.d.	2.20	3.19	7.98	Galactose-1-phosphate	1.20	0.88	0.27	0.41	0.54
Deoxyguanosine	0.81	1.12	1.00	5.97	7.58	Glucose-1-phosphate	1.20	0.88	0.27	0.41	0.54
Hippurate	n.d.	n.d.	Appear	6.78	7.38	Inositol-1-phosphate	1.20	0.88	0.27	0.41	0.54
Trimethylamine *N*-Oxide	0.80	1.13	0.78	2.83	7.33	Mannose-1-phosphate	1.20	0.88	0.27	0.41	0.54
*N*-acetylgalactosamine	1.39	1.10	1.68	5.08	6.18	Citrate	0.93	0.65	0.51	1.11	0.48
*N*-acetylglucosamine	1.39	1.10	1.68	5.08	6.18	Succinate-semialdehyde	0.76	0.66	0.58	0.75	0.47
Phenylacetylglycine	1.02	1.18	1.08	2.87	5.98	Tyrosine	0.91	0.90	0.47	0.38	0.47
Raffinose	2.38	2.92	3.04	5.51	5.89	5-Aminopentanoate	0.91	0.97	1.00	0.86	0.39
Cysteate	1.02	0.78	1.19	4.87	5.68	Threonine	0.89	0.90	0.50	0.28	0.39
Imidazole-4-acetate	n.d.	Appear	1.22	3.12	4.94	2-Aminobutyrate	0.96	0.88	0.38	0.26	0.37
Hypoxanthine	5.52	12.27	7.31	10.50	4.80	2-Aminoisobutyrate	0.96	0.88	0.38	0.26	0.37
2-Aminoadipate	Appear	2.14	4.09	3.17	4.51	Glycine	0.97	1.01	0.74	0.45	0.37
*N*-acetylglucosamine-1-phosphate	0.80	0.86	0.56	2.56	4.39	Methylthioadenosine	Disappear	n.d.	n.d.	n.d.	0.37
Glutarate	0.94	0.86	0.92	3.74	3.95	Choline	1.13	1.19	0.36	0.30	0.36
Prostaglandin E2	0.96	2.44	17.94	6.27	3.86	Adenosine	0.11	0.07	0.18	0.18	0.34
Sorbitol-6-phosphate	1.34	0.94	0.54	2.17	3.18	α-Ketoglutarate	1.47	1.01	1.20	2.60	0.33
6-Phospho-gluconate	1.79	1.17	3.05	4.51	3.15	Tryptophan	0.88	0.79	0.40	0.29	0.33
Deoxycytidine 5′-diphosphate	0.60	0.44	0.53	0.87	3.10	2,6-Diaminopimelate	1.08	0.61	0.39	0.46	0.30
Guanine	1.55	2.55	1.58	3.06	3.06	Arginine	0.93	0.67	0.20	0.11	0.30
Cytidine	1.15	0.99	0.97	1.55	2.74	Sedoheptulose-7-phosphate	1.84	1.44	0.45	0.32	0.29
*N*-acetylglucosamine-6-phosphate	Appear	1.42	2.68	5.01	2.71	Glutamic acid	0.85	0.88	0.49	0.17	0.29
*N*-acetylgalactosamine-6-phosphate	Appear	1.42	2.68	5.01	2.71	Phenylalanine	0.95	0.83	0.35	0.28	0.29
Calmodulin-like protein	n.d.	n.d.	Appear	1.95	2.64	N-Acetylornithine	0.90	0.77	0.55	0.25	0.28
Laurate	1.00	0.48	8.19	7.90	2.39	Pantothenate	1.22	1.01	0.91	0.87	0.26
3-Aminoisobutyrate	Disappear	n.d.	3.88	2.28	2.36	Leucine	0.96	0.82	0.35	0.29	0.26
3-Aminobutyrate	Disappear	n.d.	3.88	2.28	2.36	Aspartic acid	0.75	0.75	0.49	0.13	0.26
Methionine	1.16	0.83	2.11	2.67	2.13	Valine	0.95	0.91	0.43	0.31	0.24
Cytosine	1.64	1.93	1.11	1.21	2.12	Glycyl-L-leucine	0.99	0.81	0.41	0.26	0.24
2-Hydroxybutyrate	0.85	0.68	1.13	1.92	1.89	Mannose-6-phosphate	1.34	0.82	0.16	0.25	0.23
2-Hydroxyisobutyrate	0.85	0.68	1.13	1.92	1.89	Glucose-6-phosphate	1.34	0.82	0.16	0.25	0.23
Allantoate	0.96	0.87	1.02	1.90	1.84	Fructose-6-phosphate	1.34	0.82	0.16	0.25	0.23
Gluconate	1.31	1.38	2.79	7.15	1.77	Nicotinamide	0.35	0.14	0.18	0.17	0.23
Uridine diphosphate-galactose	0.31	0.33	0.57	1.20	1.77	Isonicotinamide	0.35	0.14	0.18	0.17	0.23
Uridine diphosphate-glucose	0.31	0.33	0.57	1.20	1.77	Trigonelline	n.d.	n.d.	Appear	0.70	0.22
Fructose-glutamic acid	0.98	1.12	1.41	1.58	1.73	Isoleucine	0.95	0.86	0.34	0.25	0.22
Cystathionine	n.d.	n.d.	Appear	0.84	1.70	Pyruvate	3.04	2.64	0.48	0.86	0.21
Decanoate	1.32	0.78	2.96	3.10	1.67	Glutamyl-L-alanine	0.86	0.83	0.75	0.25	0.21
Indole-3-acetic acid	n.d.	n.d.	n.d.	Appear	1.64	Pipecolate	0.96	1.04	0.33	0.28	0.19
Histidinol	0.99	1.36	5.11	3.48	1.63	*N*-Acetylglutamic acid	1.00	0.82	0.66	0.50	0.18
Nicotinamide adenine dinucleotide	n.d.	n.d.	0.71	1.15	1.60	Lactate	0.76	0.52	0.22	0.24	0.18
Galacturonate	1.23	1.17	1.27	1.61	1.60	Asparagine	0.75	0.64	0.09	0.02	0.18
Glucuronate	1.23	1.17	1.27	1.61	1.60	Glycerate	0.92	0.83	0.61	0.28	0.17
Histidine	0.82	0.69	0.79	1.46	1.54	Homoserine	1.00	0.97	0.41	0.11	0.13
Ergothioneine	0.85	0.89	0.88	1.03	1.50	Succinate	1.29	0.82	0.37	0.62	0.13
Mevalonolactone	1.14	1.31	1.19	2.12	1.38	Inosine monophosphate	1.32	0.84	0.41	0.14	0.13
Mevalonate	1.14	1.31	1.19	2.12	1.38	Adenosine diphosphate	0.14	0.14	0.19	n.d.	0.12
Pyridoxamine 5-phosphate	1.13	1.24	0.65	0.48	1.32	Guanosine monophosphate	0.95	0.68	0.39	0.16	0.11
Gibberellic acid	1.25	0.92	0.96	1.03	1.31	4-Oxovalerate	1.37	1.28	0.71	0.95	0.10
3-Hydroxybutyrate	1.01	0.97	2.45	3.31	1.22	Alanylalanine	0.74	0.98	0.48	0.12	0.09
Ornithine	1.86	2.71	1.69	1.17	1.22	5-Oxoproline	0.90	0.86	0.20	0.26	0.07
Isethionate	1.02	0.79	0.78	1.19	1.21	Adenosine monophosphate_1	0.01	0.01	0.03	0.03	0.05
Cystine	n.d.	n.d.	n.d.	Appear	1.17	Uridine monophosphate	0.81	0.52	0.23	0.09	0.05
Phenylpropanoate	1.12	1.13	1.08	1.10	1.11	Citrulline	0.78	0.74	0.32	0.08	0.03
1-Methyladenosine	0.85	0.78	0.52	0.68	1.04	β-Alanine	0.92	0.93	0.47	0.05	0.03
Glycerol-3-phosphate	1.21	1.16	0.89	1.46	1.03	γ-Aminobutyric acid	0.94	0.97	0.56	0.14	0.02
Pyridoxal	n.d.	n.d.	Appear	1.93	1.01	Homoglutamine	0.72	0.42	0.09	0.02	0.02
Fumarate	1.64	1.17	1.05	1.40	1.00	Alanine	0.93	0.89	0.22	0.04	0.02
Sulfanilic acid	1.08	0.95	0.94	1.36	1.00	Phenethylamine	0.92	0.68	0.39	0.39	Disappear
Xanthosine monophosphate	n.d.	Appear	0.87	n.d.	n.d.	Ophthalmate	4.18	3.97	0.38	Disappear	n.d.
*N*-Acetylaspartic acid	n.d.	n.d.	n.d.	Appear	n.d.	Taurolithocholate	0.79	0.42	0.38	Disappear	n.d.
Guanidinosuccinate	n.d.	n.d.	n.d.	n.d.	Appear	Diethanolamine_1	0.91	0.99	Disappear	n.d.	n.d.
*N8*-Acetylspermidine	n.d.	n.d.	n.d.	n.d.	Appear	*N*-Formylmethionine	0.81	0.63	Disappear	n.d.	n.d.
Ribulose-5-phosphate	n.d.	n.d.	n.d.	n.d.	Appear	Cytidine diphosphate	0.33	0.22	Disappear	n.d.	n.d.
Ribose-5-phosphate	n.d.	n.d.	n.d.	n.d.	Appear	Guanosine diphosphate-mannose	0.22	Disappear	n.d.	n.d.	n.d.
Xylulose-5-phosphate	n.d.	n.d.	n.d.	n.d.	Appear	Uridine diphosphate	0.11	Disappear	n.d.	n.d.	n.d.
*N*-Acetylmuramate	1.44	1.15	0.86	1.20	0.99	Guanosine diphosphate-glucose	0.22	Disappear	n.d.	n.d.	n.d.
Malate	1.06	0.78	0.93	1.50	0.96	S-adenosylhomocysteine	Disappear	n.d.	n.d.	n.d.	n.d.

n.d.: not detected, GalNAc: *N*-acetylgalactosamine, GlcNAc: *N*-acetylglucosamine, NAD: nicotinamide adenine dinucleotide, GDP: guanosine diphosphate.

## Data Availability

The datasets used and/or analyzed during the current study are available upon request from the corresponding authors due to privacy.
